# Contrasting effects of egg size and appearance on egg recognition and rejection response by Oriental reed warblers

**DOI:** 10.1002/ece3.6707

**Published:** 2020-09-01

**Authors:** Donglai Li, Xiaoshuang Li, Yan Zhang, Shuang Guan, Yanan Ruan

**Affiliations:** ^1^ School of Life Sciences Liaoning University Shenyang Liaoning China; ^2^ Present address: Changchun Longjia International Airport Changchun Jilin China

**Keywords:** brood parasitism, coevolution, common cuckoo, egg rejection, nest desertion, Oriental reed warbler, visual signal

## Abstract

**Background:**

Among potential hosts, the rejection of foreign eggs, which is a common and effective strategy to counter brood parasitism, depends on egg recognition. Multimodal and multicomponent recognition cues of brood parasitic eggs, which include both tactile (size, shape, and texture) and visual (size, shape, color, and maculation) cues, are potentially involved in the perception and discrimination of foreign eggs by hosts. An egg rejection experiment on the host with different types of model eggs can help to accurately assess the relative contribution of different components on egg recognition and constraints to rejection, in which videos can help identify the method of host rejection.

**Methods:**

Here, we assessed egg recognition and rejection responses by Oriental reed warblers (*Acrocephalus orientalis*), one of the most common hosts of common cuckoos (*Cuculus canorus*) which breed in eastern China. We designed six groups of model eggs for rejection experiments in which sensory cues included three grades of size and two categories of visual mimicry.

**Results:**

Our experiments confirmed that the multimodal traits, which included variation in size, were significant predictors of egg rejection: We detected significantly higher rejection rates of mimetic spotted model eggs than of nonmimetic blue eggs. However, large model eggs did not yield higher rejection rates and, instead, these were less likely to be rejected and more likely to be deserted compared with smaller eggs. Further video‐recording data showed that there was no significant effect of egg size on the egg recognition rate (percentage of nests with evidence of egg pecking). No evidence that the egg appearance had an effect on the method of egg rejection (ejection or nest desertion) was found.

**Conclusions:**

Only visual signals, such as color and maculation, contributed to the recognition of foreign eggs by Oriental reed warblers as recognizable clues, but not the egg size. The egg size had an impact on the type of egg rejection. It was less feasible for the warblers to eject large eggs and that is why they opted more often for desertion as the mean of model egg rejection. The significantly lower egg rejection rate of large eggs suggested that although some of them were recognized as foreign eggs, hosts failed to reject these eggs and finally the eggs were assumed to being accepted by the commonly used nest‐checking methods.

## Graphical abstract

The relative contribution of egg size and appearance (color and maculation) on egg recognition and rejection of Oriental reed warblers were studied. We detected significantly higher rejection rates of mimetic spotted model eggs than of nonmimetic blue eggs. Large model eggs did not yield higher rejection rates, and instead, these were less likely to be rejected and more likely to be deserted compared with smaller eggs.

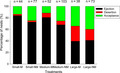

## 1 INTRODUCTION

The coevolutionary interaction between avian brood parasites and their hosts has provided an excellent set of systems to track variation in morphological evolution of parasitic eggs and egg rejection by hosts (Davies & de Brooke, 1988; Honza & Cherry, 2017; Soler, 2014; Stoddard & Stevens, 2011). In these systems, obligate brood parasitic birds lay their eggs in nests of other species (hosts), stealing partial or full parental care of the hosts, and exerting high selection pressure on the hosts to resist or decrease the negative effects of brood parasitism (Davies, 2000). One of the most important, effective, and commonly used antiparasitic strategies is to recognize and to reject foreign egg(s) (Davies & de Brooke, 1989; Langmore et al., 2005; Moksnes et al., 1991). In turn, this selection pressure forces the parasite to evolve a series of behavioral adaptations (e.g., rapid egg laying) and variations of egg morphology (e.g., egg color mimicry) to better counter egg rejection by hosts (Davies, 2011).

Although existing evidence suggests that the host uses behavioral signals, such as witnessing a parasitic event, to elicit the egg rejection action at parasitized nests (Fenney, Welbergen, & Langmore, 2012; Guigueno & Sealy, 2011; Moskát & Honza, 2002), the perceived difference in egg morphology between the eggs of parasites and hosts is thought to be the main trait for rejection decisions by hosts (Aviles, Soler, Soler, & Møller, 2004; Cherry, Bennett, & Moskát, 2007; Moskát et al., 2010; Wang et al., 2016). The eggshells of avian brood parasites generate multimodal and multicomponent recognition cues, with both tactile (size, shape, and texture) and visual (size, shape, color, and maculation) modalities potentially involved in egg perception and discrimination by hosts (Hanley et al., 2019; Honza & Cherry, 2017; Moskát, Székely, Cuthill, & Kisbenedek, 2008). However, the relative contribution of egg size and eggshell traits (i.e., color and maculation) on egg recognition by hosts and/or rejection exhibited large variations across different brood parasitic species and systems (Guigueno, Sealy, & Westphal, 2014; Segura, Di Sallo, Mahler, & Reboreda, 2016; see reviews in Honza & Cherry, 2017).

Rothstein (1982) conducted an early experiment on American robins (*Turdus migratorius*) to assess the relative effects of size, color, and maculation on egg recognition by hosts. He found that variation in these three features had the same directional effects and that birds rejected model eggs that differed by at least two of these three features from the robin's own eggs. This experiment was further replicated by Luro et al. (2018), which confirmed most of the original findings, but also showed that variation in egg size was less predictive than the other two features. Guigueno et al. (2014) conducted a comprehensive review on this topic and further confirmed that color was the most important cue that hosts used to recognize and to reject parasitic eggs, especially for hosts of the common cuckoo (*Cuculus canorus*) (Antonov, Stokke, Moksnes, & Røskaft, 2006; Stokke et al., 2010). However, in their experimental study in yellow warblers (*Dendroica petechia*), which is a host of brown‐headed cowbirds (*Molothrus ater*), Guigueno et al. (2014) found that egg size was the primary cue for hosts that use egg burial or nest desertion as a rejection method. Indeed, other studies implied that egg size was the primary parameter used for ejection by some ground nesting (Marchetti, 2000) or dome nesters (Langmore, Hunt, & Kilner, 2003) because color was less visible in dark nest environments.

In addition to the cues of cognitive recognition, egg size and eggshell appearance (i.e., color and maculation) might represent different traits that were involved in the egg rejection response (ejection, desertion, or burying). A large number of studies have confirmed that hosts use cues from egg coloration or maculation mimicry to reject eggs (Honza & Cherry, 2017; Šulc, Procházka, Capek, & Honza, 2016), but evidence for using egg coloration as an egg rejection cue is rare (Šulc et al., 2019). Egg size can significantly affect the mode of egg rejection by forcing hosts to desert nests instead of ejecting eggs if they are constricted by their limited bill size or the weight of a heavy parasitic egg (Stokke et al., 2010; Roncalli, Ibáñez‐Álamo, & Soler, 2017; but see Soler, Ruiz‐Raya, Roncalli, & Ibáñez‐Álamo, 2015). Additionally, hosts may accept a parasitic egg sometimes, even if it has been recognized (i.e., evidence of egg‐pecking behavior from video sampling) (Guigueno & Sealy, 2012; Soler, Fernández‐Morante, Espinosa, & Martín‐Vivaldi, 2012). This phenomenon has been recorded in several small‐bodied hosts (about 10 g; Roncalli et al., 2017), especially in experiments using artificial model eggs made from a hard material (Martín‐Vivaldi, Soler, & Møller, 2002; Šulc et al., 2016). However, this effect is thought to be less constrained in large or medium‐sized hosts that grasp or puncture foreign eggs in their nests (Soler, Ruiz‐Raya, Roncalli, & Ibáñez‐Álamo, 2017).

We studied the Oriental reed warbler (*Acrocephalus orientalis*), a frequently parasitized host of common cuckoos in eastern China (Li et al., 2016a, 2016b; Yang, Wang, Liang, & Møller, 2015, 2017). We designed six groups of model eggs that differed in size (small, medium, large) and/or in color and maculation (nonmimetic: immaculate blue; mimetic: pale blue background color and brown spots) to conduct egg rejection experiments. We hypothesized that (a) the mimetic spotted egg model should be recognized and rejected less often than the blue nonmimetic egg; (b) if egg size serves as a recognition cue, model eggs should be rejected also based on their differences from host eggs (i.e., large and small eggs should be recognized and rejected more often than medium eggs). Otherwise, if egg size of the model egg constrains egg ejection by Oriental reed warblers, we hypothesized that (c) it should have a higher rate of nest desertion for large model eggs compared with smaller eggs. To confirm the egg rejection responses and to assess whether all recognized eggs were rejected, we filmed at nests to compare the variation in the perceived recognition (i.e., egg pecking) and final egg rejection responses (ejection or desertion). In addition, effects of egg color or size on egg‐pecking behavior and on successful ejection (or desertion) during the 8‐hr video‐recording time were also examined.

## 2 METHODS

### 2.1 Study site and species

This study was conducted during 2010–2012 and 2013–2018 in the Yellow River Delta, Shandong Province, and the Liaohe River Delta, Liaoning Province, China, respectively. The distance between these two sites was 400 km. Both sites were typical estuarine wetlands and contained a large area of reed field, a common breeding habitat for Oriental reed warblers and common cuckoos in China (Li, Zhang, Grim, Liang, & Stokke, 2016). The mean clutch size of Oriental reed warblers was 4.74 ± 0.52 (*SD*, *n* = 276) and varied from four to six eggs (Li, 2012). Cuckoo parasitism rates across the two sites varied between 17% and 25%. Detailed information about the study sites can be found in Li, Ruan, et al. (2016), Li, Zhang, et al. (2016).

### 2.2 Experimental design, model egg production, and egg reflectance measure

**FIGURE 1:**
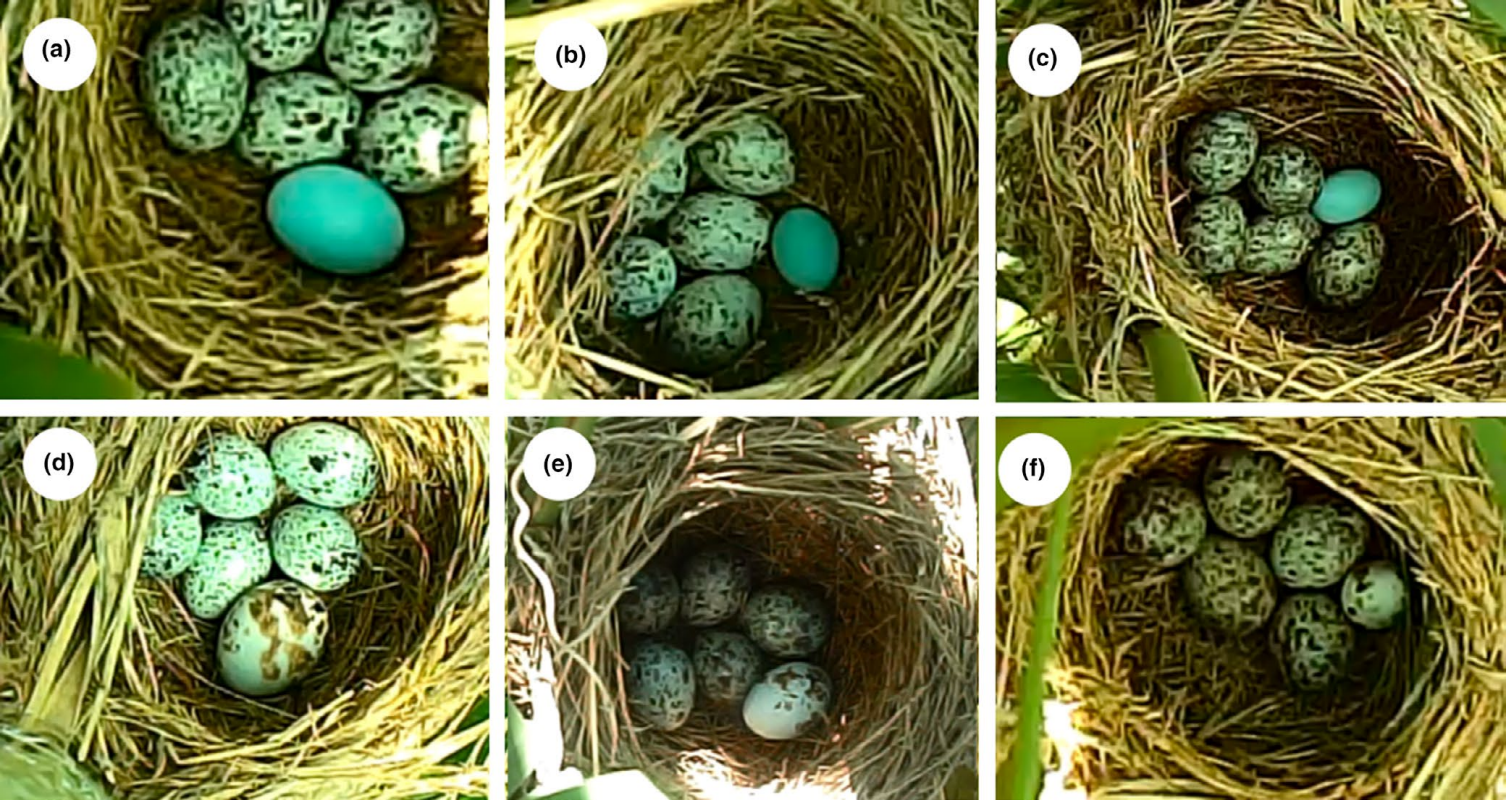
Experimental egg models used in Oriental reed warbler nests to test the effects egg size, color, and spotting on egg rejection; large (a), medium (b), and small (c) size blue, nonmimetic eggs, and large (d), medium (e), and small (f) spotted mimetic eggs

**FIGURE 2:**
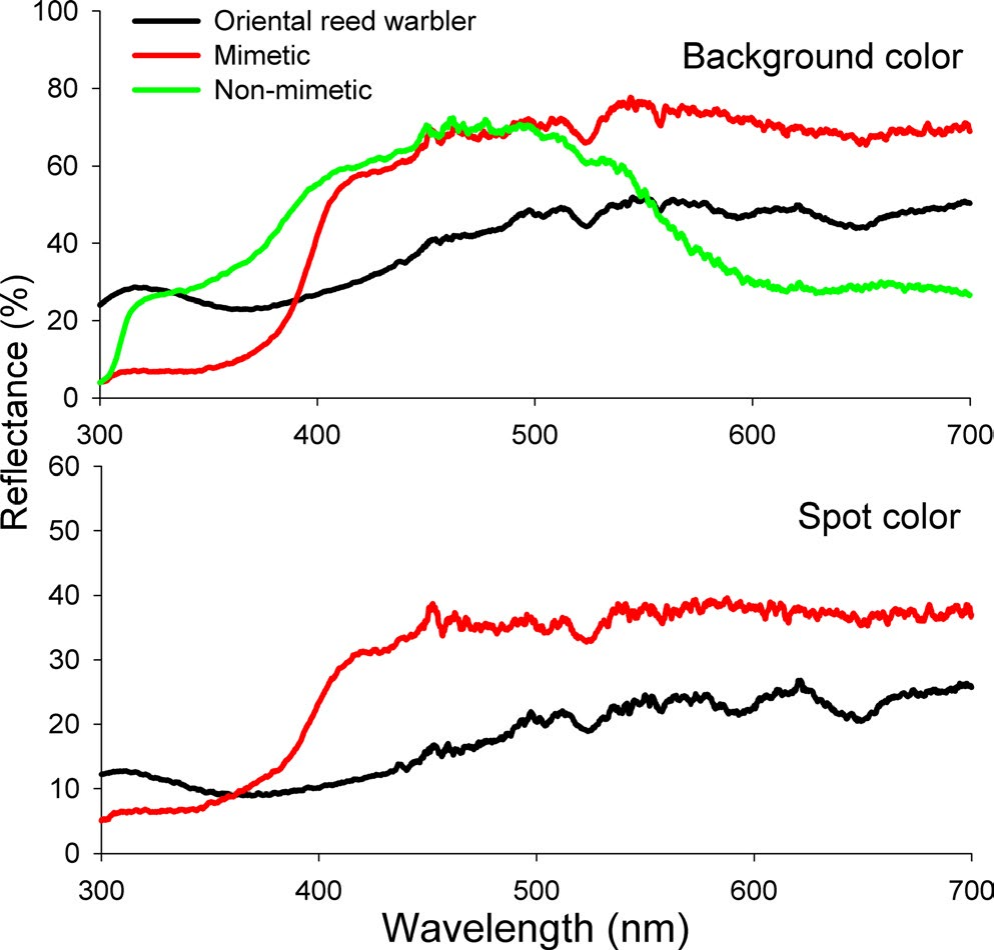
Comparison of reflectance spectra among nonmimetic blue egg models, mimetic egg models, and natural (real) Oriental reed warbler eggs with a solid background color (above) and spotted (below). The spectra reflectance of the color of mimetic egg models shows a pattern more similar to Oriental reed warbler eggs than nonmimetic egg models, although there were different contrasts between the UV (300–40 nm) and human vision (400–700 nm) wavelengths

We used a polymer clay (Ai Tao Le, Shenzhen, China) to produce the model eggs rather than using painted real eggs in the experiment because it was difficult to find a sufficient number of natural eggs of different sizes. We designed six groups of model eggs with two different colors and spotting and with three different sizes (Figure 1). The nonmimetic model egg was pale blue, and the white model egg was painted with a light blue‐green background (Sakura, Japan; #236) with dark brown spots (#17) to mimic the true Oriental reed warbler eggs (Figure 1). The medium size of model eggs was similar to local Oriental reed warblers egg size (21.32 mm × 15.51 mm, *n* = 70 clutches), large eggs were 67% larger than medium eggs, and small eggs were 50% smaller than medium eggs (Table 1). Egg reflectance spectra (300–700 nm) of the background color and spot color of model eggs and Oriental reed warbler eggs were measured using a miniature fiber optic spectrometer (AVANTES) following the protocol of Li, Zhang, et al. (2016) (Figure 2).

**TABLE 1:** Egg phenotypes (mean ± *SD*) of three different‐sized egg models used in the egg rejection experiments on Oriental reed warblers in eastern China

Egg types	Mass (g)	Egg length (mm)	Egg breadth (mm)	Volume (cm^3^)
Large size model eggs (*n* = 20)	5.22 ± 0.08	24.66 ± 0.80	17.65 ± 0.41	3.91 ± 0.12
Medium size model eggs (*n* = 20)	3.20 ± 0.03	20.84 ± 0.48	14.85 ± 0.33	2.34 ± 0.01
Small size model eggs (*n* = 20)	1.61 ± 0.03	16.96 ± 0.61	11.63 ± 0.22	1.17 ± 0.04
Common cuckoo eggs (*n* = 28)	3.16 ± 0.27	21.90 ± 0.88	16.25 ± 0.41	2.96 ± 0.25
Oriental reed warbler eggs (*n* = 70)	2.62 ± 0.24	21.32 ± 0.84	15.51 ± 0.41	2.62 ± 0.20

Note

Egg length and breadth were measured with a digital caliper to 0.01 mm, and the weight was measured with electronic balance (0.01 g). We calculated egg volume (cm^3^) as 0.51 * Length * Breadth2 * 1,000 (Hoyt, 1979). The values of Oriental reed warbler eggs were averaged per clutch.

### 2.3 Rejection experiments

Each breeding season, reed beds were searched systematically for Oriental reed warbler nests. The number of eggs (empty nest as 0), incubation stage, and whether the nest was parasitized were recorded the first time it was found and when rechecked every 1–3 days. Oriental reed warblers usually lay five eggs and begin incubation after laying the penultimate egg (Li et al. unpublished data). The stage of embryo development was assessed by candling if the nest was found after clutch completion (Weller, 1956). Those nests that were close to hatching with an obvious embryo were not used in this experiment. The clutch initiation date was determined by counting eggs (assuming one egg was laid per day and that no eggs were lost) or calculated when the chick hatching date was recorded; eggs usually needed 12 days to complete incubation. Most cuckoos typically parasitize the warblers during the laying stage (Lotem, Nakamura, & Zahavi, 1995; Wang, Yang, He, Liang, & Møller, 2020) and only nests that did not contain cuckoo eggs were used for egg recognition experiment. As a result, the onset of egg experiment for each nest was divided into three stages: (a) egg‐laying stage: when the females rarely visited their nests, (b) early incubation stage: ≤4 days after clutch completion, and (c) late incubation stage: 5–8 days after clutch completion.

One of the six types of model eggs was introduced randomly into Oriental reed warbler nests and checked every other day until the 6th day (Bártol, Karcza, Moskát, Røskaft, & Kisbenedek, 2002; Moksnes et al., 1991). We added the egg model without removing any host eggs. Several studies confirmed that this is a normal procedure for this kind of experiment, which does not alter the rejection rate (Davies & de Brooke, 1989; Grim & Honza, 2011). Only nonmimetic model eggs (sample sizes for the small, medium, and large eggs were 33, 40, and 24, respectively) were trialed in Yellow River Delta, and the egg rejection rates of these model eggs were not significantly different from those in Liaohe River delta (chi‐square tests: all *p* values ≥ .212 for three egg sizes).

When the model egg disappeared from the nest and the warbler eggs were still warm, it was classified as an ejection; when the model egg was still in the nest, but the warbler eggs were cool during two times of nest checking, it was classified as desertion. Both ejection and desertion were pooled as rejection. Otherwise, the model egg in the nest was rechecked if there was a trace on it, which was presumed to form during pecking or piercing attempts made by a host; this was an example of “egg recognition,” but it was not included as “rejection” because it was not rejected successfully (i.e., either ejected or deserted). Only nests where the model egg showed no trace of pecking and were still being incubated were recognized as accepted. We found model eggs buried in nest material in only two nests.

### 2.4 Video surveillance of host nests

Another subset of nests (*n* = 76) was filmed using Xiaoyi 4K digital cameras (Shanghai, China) for 8 hr after the introduction of a model egg during 2014–2018. In addition, we selected another 13 nests randomly in which we marked one egg with a water‐proof pen as controls. We also monitored these nests for 6 days to determine the host response.

We checked the videos after the experiment using Baofeng 5.0 digital player. Similar to the closely related Eurasian great reed warbler (*Acrocephalus arundinaceus*). Oriental reed warblers are “puncture ejectors” that usually peck foreign eggs strongly (Honza & Moskát, 2008; Požgayová, Procházka, Polačiková, & Honza, 2011), considering that their grasp index (161.5 ± 15.7 mm^2^; *n* = 21; Li, 2012) were less than the supposed values to be grasper ejectors (200 mm^2^; Rohwer & Spaw, 1988). If the warbler strongly pecked the model egg repeatedly using its bill, it was classified as “egg pecking,” which indicated that the warbler “recognized” the experimental egg. The percentage of nests with evidence of “egg pecking” was summed for each egg type. To have an accurate time of egg recognition, we calculated the initial time to egg pecking (h) since the time when the host first appeared at the nest. In addition, egg‐pecking frequency (/h) before the model egg was rejected was also counted from the video.

### 2.5 Statistical analyses

In total, we experimentally parasitized 404 nests of Oriental reed warblers with model eggs. Three types of egg rejection behaviors were identified at 250, 40, and two nests, where foreign eggs were ejected, deserted, and buried, respectively. Another 15 model eggs were pecked with marks, but not ejected successfully during the first six days. All of the nests with ejection, desertion, egg pecking, and egg burial were included in the “egg recognition” dataset to test for a cognitive response (binomial, 1 = recognized, 2 = not recognized; *n* = 404). All other nests, except for the two buried nests and 15 “pecked” nests, were used for the “egg rejection” dataset to test for the rejection rate (binomial, 1 = egg rejection, 2 = acceptance; *n* = 389). The two buried nests were not included in the dataset for egg rejection because it was a rarely used rejection method. There was no difference in the statistical conclusion whether these two nests were considered as rejecters or not. In addition, to test the behavioral response of egg rejection, all nests classified as ejection or desertion were pooled as the “rejection response” (binomial, 1 = egg ejection, 2 = desertion; *n* = 290) dataset for model building.

Three generalized linear mixed models (GLMM: binomial error and logit link function) were fitted by maximum likelihood (Laplace approximation) with the “egg recognition,” “egg rejection,” and “rejection response” as response variables using the *lme4* package (version 1.1‐21; Bates et al., 2019). The two traits of model eggs, egg color (nonmimetic pale blue eggs and mimetic spotted eggs) and egg size (small, medium, and large) and their interaction were included as categorical predictors in all models. Other potential explanatory predictors included clutch size (the number of eggs inside the nest when experimental model eggs were introduced), clutch initiation date, and incubation stage (1: egg‐laying stage; 2: early incubation stage; 3: late incubation stage). Because there were no special hypotheses for variation among years for egg rejection, the year was included as the random factor. No evidence of collinearity among variables was found when tested using the variance inflation factor (VIF), which was <2 for all of them (Zuur, Leno, & Elphick, 2010).

We used R 3.6.0 with RStudio‐1.2.1335 (RStudio, Inc, Boston, MA) for statistical analyses. Global models were run first and then standardized (*z*‐scores) for all predictors using the standardize function in the *arm* package (version 1.10‐1). Then, all candidate models were generated from global models using the dredge function in the *MuMIn* package 1.43.6 (Barton, 2019). We used Akaike's information criteria (AICc; Burnham & Anderson, 2002) corrected for low sample sizes to assess model fit. All models were ranked using ΔAIC values, and models with ΔAIC ≤ 2 were considered as equivalent alternative models. Akaike weights (w_i_) were used to provide a quantitative measure of support for each model relative to the others. Conditional model‐averaged parameter values (*β*‐values) from all equivalent models were generated by the model averaging function in the *MuMIn* package. Wald test *Z*‐scores were explored to make inferences about each parameter estimate. The significance of parameters was also indicated with the profiled 95% confidence interval (CI), which did not contain zero.

For the 76 video‐monitored nests, we used linear mixed models (LMM) to explore the effects of egg color and size on the time to egg pecking (h) and egg‐pecking frequency (/m), separately, with Gamma error and log link function. We built our optimal model by including the following predictors: egg color, egg size, clutch size, incubation stage, and clutch initiation date. The year was included as the random factor. During backward elimination stepwise model selection fitted by restricted maximum likelihood (REML), nonsignificant terms were dropped and, as a result, the optimal model was shown in the results. For these variables, the significance of effects was assessed from Satterthwaite's *t* test for models by using the *lmtest* package. The variations in egg recognition rate (percentage of nests with evidence of egg pecking) among group and the difference between egg recognition rate and egg rejection rate (egg ejection and desertion) were compared using chi‐square tests. Mann–Whitney *U* tests were used to compare the differences in the latency to ejection and number of incubation bouts before ejection between medium and small eggs. The alpha threshold was set to 0.05, and the results were expressed as the mean ± standard error, unless stated otherwise.

## 3 RESULTS

### 3.1 Egg rejection rate and the rejection types

For the “egg recognition” dataset that included all the nests ejected, deserted, buried, or model eggs with peck marks, the analyses yielded two equivalent alternative models (Table 2). In contrast to hypothesis 1, the pale blue nonmimetic eggs were recognized by hosts significantly less than mimetic spotted eggs (effect estimation: *β* = −0.72 ± 0.31; *z* = 2.314, *p* = .021), although we had predicted it would be higher. In addition, the large model eggs were recognized significantly less than medium eggs (*β* = −0.79 ± 0.29; *z* = 2.703, *p* = .007); no significant difference was detected between small eggs and medium eggs (*β* = 0.04 ± 0.33; *z* = 0.122, *p* = .903; Table 3), which did not fit hypothesis 2. No other confounding predictors, which included stage of incubation, had a significant effect on the model explanation (Table 3).

**TABLE 2:** Candidates of the model selection approach using the GLMM models with the response (recognition, rejection, and ejection) as dependent values

Response variables	Models	*df*	AICc	Delta AICc	Weight
recognition	color+size+incubation	8	439.01	0	0.53
	color+size+incubation+clutch size	9	439.22	0.21	0.47
rejection	color+size+incubation	8	426.66	0	0.49
	color+size+incubation+clutch size	9	427.4	0.74	0.34
	color+size+incubation+clutch initiation date	9	428.65	1.99	0.18
ejection (vs. desertion)	size+incubation	7	201.91	0	0.32
	size	5	203.36	1.45	0.16
	size+incubation+clutch initiation date	8	203.57	1.66	0.14
	size+clutch size	6	203.65	1.74	0.13
	size+incubation+clutch initiation date	8	203.7	1.78	0.13
	color+size+incubation	8	203.89	1.98	0.12

Note

The models were chosen from among successive candidate models using the criterion of ΔAIC ≤ 2, and the models were ranked with Akaike weights (wi). The reference categories for “color,” “size,” and “incubation” are “mimetic,” “medium,” and “egg‐laying stage,” respectively.

**TABLE 3:** Model‐averaged coefficients from the GLMM candidates in the response to egg recognition rate, egg rejection rate, and the rejection responses (ejection compared with desertion)

Response variables	Parameters	Estimate	Std. error	95% confidence interval		*z* value	*p* value	Relative importance
				Lower	Upper			
Recognition (recognized vs. not recognized)	(Intercept)	1.92	0.44	1.07	2.78	4.408	**<.001**	
	**color:nonmimetic**	**−0.72**	**0.31**	**−1.33**	**−0.11**	**2.314**	**.021**	**1**
	incubation: early	0.4	0.41	−0.41	1.21	0.96	.337	1
	incubation: late	−0.67	0.44	−1.54	0.21	1.493	.136	
	size: small	0.04	0.33	−0.61	0.69	0.122	.903	1
	**size: large**	**−0.79**	**0.29**	**−1.37**	**−0.22**	**2.703**	**.007**	
	clutch size	−0.58	0.42	−1.41	0.26	1.354	.176	0.47
Rejection (acceptance vs. rejection)	(Intercept)	1.97	0.45	1.09	2.84	4.41	**<.001**	
	**color:nonmimetic**	**−0.74**	**0.33**	**−1.39**	**−0.1**	**2.264**	**.024**	**1**
	incubation: early	0.37	0.38	−0.37	1.12	0.974	.33	1
	incubation: late	−0.76	0.42	−1.59	0.07	1.787	.074	
	size: small	0.04	0.34	−0.62	0.71	0.13	.896	1
	**size: large**	**−0.94**	**0.3**	**−1.53**	**−0.35**	**3.102**	**.002**	
	clutch size	−0.49	0.42	−1.33	0.34	1.152	.249	0.34
	clutch initiation date	0.08	0.26	−0.43	0.6	0.318	.75	0.18
Rejection type (ejection vs. desertion)	(Intercept)	4.05	0.87	2.33	5.77	4.611	**<.001**	
	color:nonmimetic	0.25	0.67	−1.07	1.56	0.369	.712	0.12
	incubation: early	−0.93	0.54	−1.99	0.14	1.703	.089	0.71
	**incubation: late**	**−1.56**	**0.77**	**−3.07**	**−0.06**	**2.033**	**.042**	
	size: small	1.12	0.71	−0.29	2.52	1.559	.119	1
	**size: large**	**−1.78**	**0.47**	**−2.7**	**−0.86**	**3.799**	**<.001**	
	clutch size	−0.09	0.76	−1.59	1.41	0.117	.907	0.27
	clutch initiation date	0.32	0.55	−0.77	1.41	0.581	.561	0.14

Note

Statistically significant parameters, parameter estimates, and standard errors (*SE*s) are highlighted in bold. The reference categories for “color,” “size,” and “incubation” are “mimetic,” “medium,” and “egg‐laying stage,” respectively.

**FIGURE 3:**
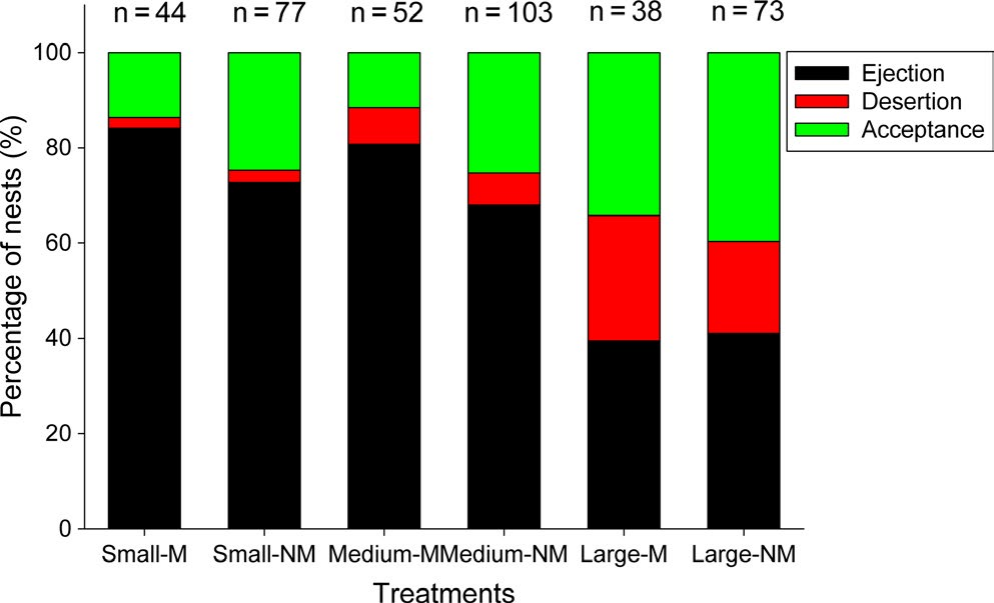
Host responses to experimental egg models by the Oriental reed warblers. The 15 nests where eggs were pecked at but not ejected and two deserted nests were not included

For the “egg rejection” dataset, both egg color and egg size were the main contributing predictors with the same relative importance in the top three GLMM models based on the model selection (Table 2). The egg rejection rate of pale blue nonmimetic eggs was significantly lower than for mimetic spotted eggs (*β* = −0.74 ± 0.33; *z* = 2.353, *p* = .019). There were significantly lower egg rejection rates in response to the large model eggs relative to medium eggs (*β* = −0.94 ± 0.30; *z* = 3.102, *p* = .002), but there was no significant difference between small eggs and medium eggs (*β* = 0.04 ± 0.34; *z* = 0.130, *p* = .896; Figure 3). There was also marginally significantly lower egg rejection rate in the late incubation stage than in the egg‐laying stage (model average: *β* = −0.76 ± 0.42; *z* = 1.787, *p* = .074), but no statistical difference between early incubation and the egg‐laying stage (Table 3). No other confounding predictors, which included the interaction between egg color and size, had a significant term to contribute to the model (Table 3).

For the egg rejection response (ejection compared with desertion), only egg size was included in all the six top models (Table 2), which implied a significant contribution of egg size to the model's explanatory power (relative importance value: 1). As predicted by hypothesis 3, large model eggs were less likely to be ejected than medium eggs (*β* = −1.78 ± 0.47; *z* = 3.799, *p* < .001), but large model eggs were deserted more often than medium eggs (Figure 3). There was no significant difference in the ejection rate between small eggs and medium eggs (*β* = 1.12 ± 0.71; *z* = 1.559, *p* = .119) and other potential predictors, which included egg color (*β* = 0.25 ± 0.67; *z* = 0.369, *p* = .712). However, there was a significantly lower ejection rate later in incubation than in the egg‐laying stage (*β* = −1.56 ± 0.77; *z* = 2.033, *p* = .042; Table 3).

### 3.2 Video monitoring of the host responses (egg rejection compared with egg pecking)

**FIGURE 4:**
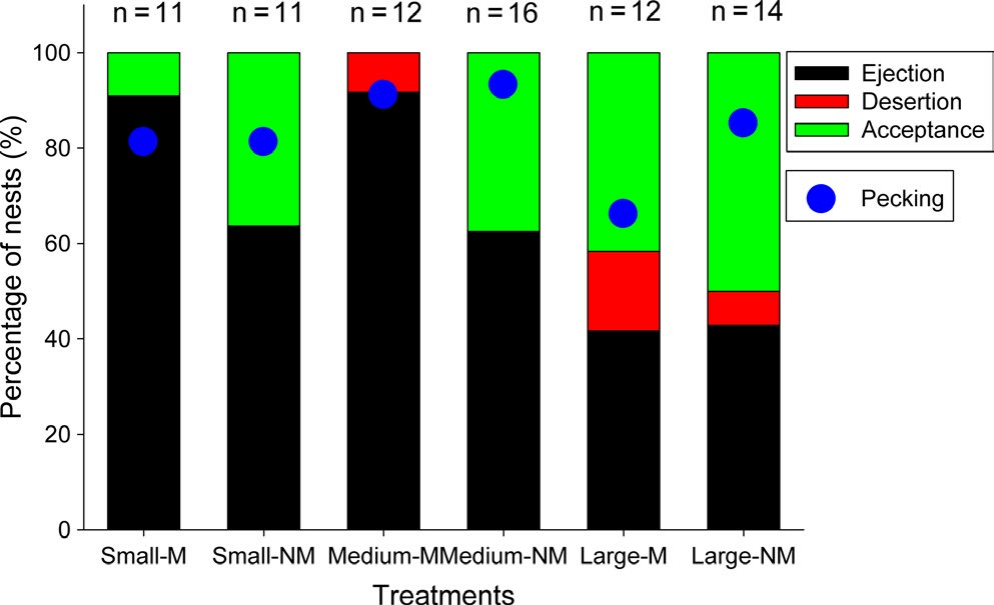
Comparison of egg rejection and egg recognition (egg pecking) by Oriental reed warblers to six types of egg models that differed in mimicry and size. Nests were monitored for 8 hr through video recording and across the typical 6‐day nest checking

In total, 69.7% (*n* = 76) of model eggs introduced into the video‐monitoring nests were rejected successfully. Meanwhile, “egg‐pecking” behavior was recorded in 84.8% of nests, which approached significantly higher than the actual rejection rate (χ^2^ = 3.549, *df* = 1, *p* = .06). Specially, there were relatively higher “egg‐pecking” rates (percentage of nests with evidence of egg pecking) than the egg rejection rates for the two color types of large model eggs (Figure 4) though not statistically significant (χ^2^ = 2.214, *df* = 1, *p* = .145); this confirmed that large model eggs were difficult to reject even though they were recognized as foreign eggs. In turn, for medium and small eggs, the recognition rates approached significantly higher than rejection rate for nonmimetic eggs (χ^2^ = 3.647, *df* = 1, *p* = .056), but not for the mimetic eggs (χ^2^ = 0.247, *df* = 1, *p* = .601).

**FIGURE 5:**
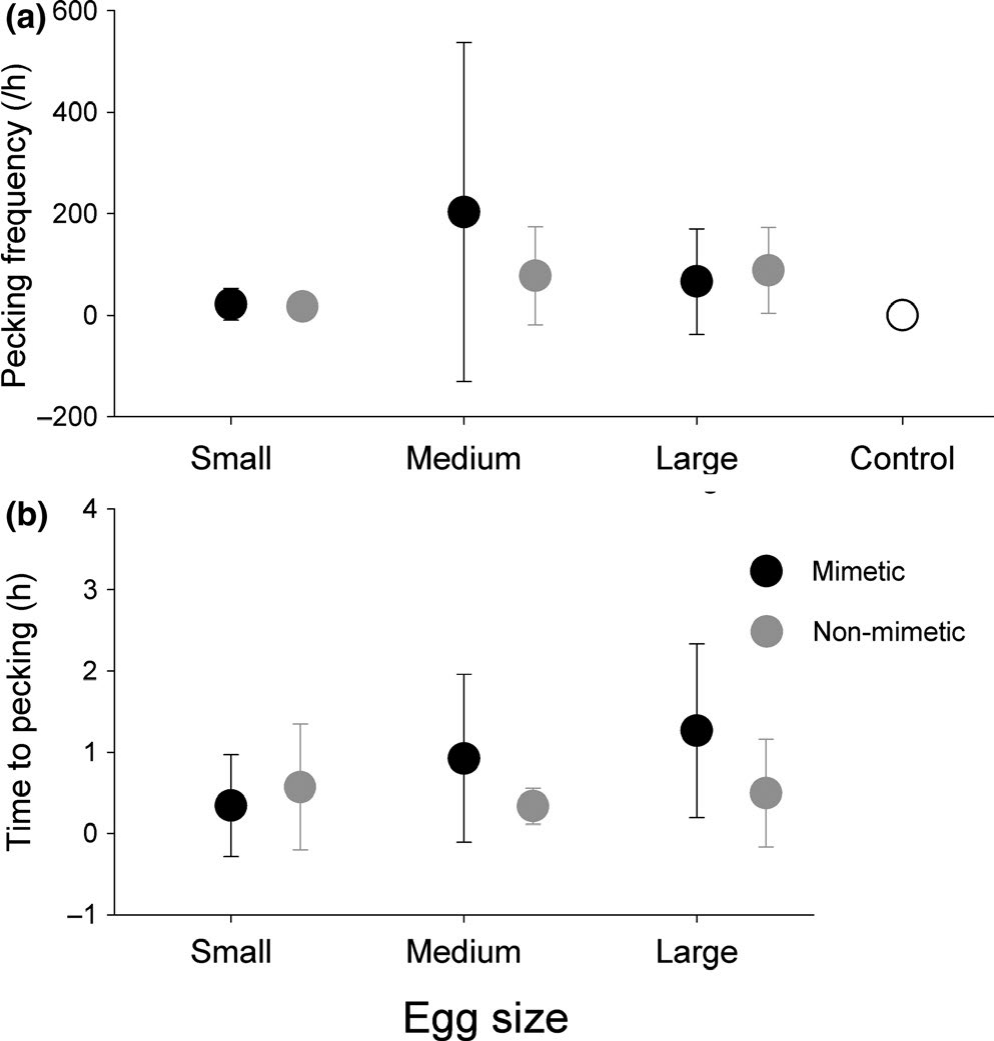
Comparison of the mean egg‐pecking frequency and time to egg pecking since the first incubation attempt in Oriental reed warblers among six types of artificial parasitized and control nests. The error bars represent the 95% confidence interval

None of the marked eggs in control nests were pecked. There were no significant differences in the “egg‐pecking” rate among size (χ^2^ = 2.708, *df* = 2, *p* = .258) and egg color (χ^2^ = 0.378, *df* = 1, *p* = .539). There were also no significant effects of egg color or size on the egg‐pecking frequency (/h) and time to egg pecking (h) in the experimentally parasitized nests. However, the warblers had significantly higher egg‐pecking frequencies of medium eggs than small eggs (*β* = 0.38 ± 0.15; *t* = 2.501, *p* = .015; Figure 5). No significant effects of clutch size and clutch initiation date were found in either of the two models (Table 4).

**TABLE 4:** Estimated coefficients of egg color and size from the maximal model of LMM fitted by REML in response to egg‐pecking frequency and time to egg pecking

Responsive variables	Parameters	Estimate	Std. error	95% confidence interval		*t* value	Pr(>|*t*|)
				Lower	Upper		
Pecking frequency (/h)	(Intercept)	−0.31	0.25	−0.77	0.16	−1.244	0.218
	color:nonmimetic	−0.02	0.12	−0.24	0.20	−0.160	0.874
	**size: small**	**−0.38**	**0.15**	**−0.66**	**−0.09**	**−2.501**	**0.015**
	size: large	−0.25	0.14	−0.52	0.02	−1.741	0.086
	incubation: early	0.10	0.24	−0.35	0.54	0.404	0.688
	incubation: late	0.29	0.27	−0.22	0.81	1.068	0.289
	clutch size	0.06	0.16	−0.24	0.35	0.359	0.721
	clutch initiation date	0.21	0.13	−0.04	0.46	1.557	0.124
Time to pecking (h)	(Intercept)	−0.02	0.28	−0.50	0.52	−0.057	0.955
	color:nonmimetic	−0.23	0.13	−0.45	0.04	−1.729	0.090
	size: small	−0.01	0.16	−0.32	0.28	−0.069	0.945
	size: large	0.15	0.17	−0.18	0.45	0.895	0.374
	incubation: early	0.11	0.28	−0.43	0.57	0.390	0.699
	incubation: late	0.06	0.31	−0.52	0.63	0.184	0.855
	clutch size	−0.17	0.18	−0.47	0.20	−0.936	0.353
	clutch initiation date	−0.17	0.16	−0.44	0.20	−1.024	0.312

Note

Statistically significant parameters, parameter estimates, and standard errors (*SE*s) are highlighted in bold. The reference categories for “color,” “size,” and “incubation” are “mimetic,” “medium,” and “egg‐laying stage,” respectively.

No nests were deserted during 8 hr of video‐recording time, and no large model eggs (*n* = 24) were ejected successfully. On the contrary, 14 nests of medium (21.4%, *n* = 28) and small size (36.4%, *n* = 22) model eggs were recorded to be ejected in the video. Mean latency for ejection was 2.26 ± 2.74 (mean ± *SD*) h and varied from 0 to 7.92 hr; mean number of incubation bouts (per h) was 9.64 ± 10.31 (mean ± *SD*) times and varied from 1 to 33 times. There were no significant differences in either latency to ejection (Mann–Whitney *U*: *U* = 19.00, *p* = .573) or in the number of incubation bouts needed for the ejection (*U* = 18.5, *p* = .491) between small and medium model egg sizes.

## 4 DISCUSSION

Our egg recognition experiments showed a relatively high egg rejection rate for all kinds of model eggs, which included a significant variation in rejection rates based on egg size. Both the egg “recognition” and “rejection” models yielded similar statistical conclusion. This implied that multimodal cues of egg size and color, and spotting might be involved in egg perception and discrimination by hosts (Honza & Cherry, 2017; Rothstein, 1982). However, the contribution of visual signals, such as color and maculation, appeared to be greater than egg size for recognizing foreign eggs by Oriental reed warblers because the fixed factor of “egg color” served as a strong predictor in both the “recognition” and “rejection” models, but there was no significant difference in rejection rate between medium eggs and small eggs. Furthermore, the large model eggs did not yield higher rejection rates as supposed in the second hypothesis and, instead, these were less likely to be rejected compared with medium eggs. This implied that egg size was not an important cue for egg recognition, at least not for this host population with the model eggs. The result was further confirmed by video samples of the cognitive assessment of egg recognition by “pecking”: Among nests adding eggs with different sizes and the same color, there was no significant variation in the percentage of nests in which hosts showed egg‐pecking behavior (Figure 4). Additionally, there was no evidence that large and small eggs were pecked more frequently than medium eggs if egg size was used as a recognition cue. On the contrary, we found a higher pecking frequency for medium eggs than for small eggs, which might possibly be related to a sampling problem with a large 95% confidence interval for medium eggs (Figure 5a). Therefore, our results for egg rejection by Oriental reed warblers were similar to the conclusion by Antonov et al. (2006) and Luro et al. (2018) that for an egg rejecter host, egg size has relatively less effect than other visual traits of the eggshell.

Although there was no general evidence of egg size on egg recognition, the large model eggs elicited a higher percentage of nest desertion than medium or small model eggs. This supported previous findings that egg size affected the egg rejection method, but not the overall rejection rate, especially when using model eggs made from hard materials (Martín‐Vivaldi et al. 2002; Šulc et al., 2016). The inability of this host to pierce a model egg could lead to more nest desertion (Antonov, Stokke, Moksnes, & Røskaft, 2009). It has been suggested that the use of hard model eggs in egg rejection experiments underestimate true egg rejection rates because some of the eggs that may have been recognized were eventually accepted when the hosts found it physically impossible to eject them (Martín‐Vivaldi et al. 2012; Soler et al., 2017). Our video‐recorded nests clearly supported this viewpoint. The recognition rates for that same model egg type (i.e., for the large model egg treatments of nests where we recorded the warbler pecking the models) tended to be higher than that of the actual egg rejection rate.

Relative to the egg size effect, strictly visual signals should be viewed as the main contributing predictors in the warbler's egg rejection behavior here. Both the difference in egg color and the spotting pattern were thought to cause egg rejection, but egg color did not decrease egg rejection: The spotted model eggs appeared more mimetic to the warbler egg, at least to humans, and yet they elicited a relatively higher egg rejection rate than the immaculate “nonmimetic” blue model eggs. This result refutes the first hypothesis that the mimetic spotted model eggs should be rejected less than nonmimetic pale blue eggs. It was inconsistent with recent work showed that chalk‐browed mockingbirds (*Mimus saturninus*) rejected unspotted eggs more than spotted eggs (Peer, Ellison, & Sealy, 2002; Hanley et al., 2019; but see Liu, Yang, Yu, Wang, & Liang, 2019).

There are at least three reasons to explain why the mimetic spotted eggs were rejected more frequently than nonmimetic blue eggs. First, it may be that the imperfect mimicry of our spotted model eggs increased the difference in appearance from host eggs, especially considering that Oriental reed warblers have evolved an excellent egg rejection ability that can use minor differences between a foreign egg and their own (Li, Ruan, et al., 2016; Li, Zhang, et al., 2016; Moskát et al., 2010). Second, there may be different cognitive channels with respect to color and spot recognition contexts (Hanley et al., 2019). For example, spot pattern requires a higher cognitive signal than seeing the contrast in coloration, as suggested by the spotting pattern on each egg that has an individual identity signal, which can be used to reject foreign eggs by some hosts (Stoddard Kilner & Town 2014; Liu et al., 2019). Oriental reed warblers have experienced a long evolutionary history of cuckoo parasitism and have also evolved the ability to identify a large variety of spotting patterns in clutches (Li, Ruan, et al., 2016). This makes it more possible for them to reject the spotted mimetic model egg if it is, in fact, not mimetic of the hosts' own eggs.

Third, the nonmimetic blue model egg may actually better mimic the ultraviolet (UV) part of the spectrum of host eggs than that of the brown spotted model eggs (as shown in Figure 2 for the UV 300–400 nm wavelengths). UV light may be an important factor in recognizing parasitic eggs by various hosts (Honza & Polaciková, 2008), which include closely related reed warbler species (Šulc et al., 2016). This result was also consistent with recent reports that related reed warbler hosts rejected more model eggs reflecting long‐wave lengths of light compared to the host eggs than model eggs reflecting short‐wave lengths; that is, our pale blue nonmimetic eggs may appear more likely to the green‐blue background of the warbler's own natural eggs than the spotted eggs (Abolins‐Abols, Hanley, Moskát, Grim, & Hauber, 2019; Hanley et al., 2017, 2019; Manna et al., 2020).

Evidence of the effect of incubation stage on egg rejection was inconsistent. Individual hosts exhibited high repeatability in their egg rejection behaviors (Grim, Samas, & Hauber, 2014; Honza, Pozgayová, Procházka, & Tkadlec, 2007; Luro & Hauber, 2017; Samas, Cassey, Hauber, & Grim, 2011), and there was no effect of incubation stage on rejection (Soler et al., 2015). In contrast, we found that the egg rejection rate, particularly the ejection rate, tended to decline later in incubation in Oriental reed warblers. A similar flexible pattern across incubation stages was also reported in great reed warblers (*Acrocephalus arundinaceus*) (Moksnes et al., 1990; Moskát, 2005). The possible reason for this pattern might be related to decreased parasitism risk in the later stage of incubation, and an endocrine‐related motivational shift for egg rejection, which deserves more research (Abolins‐Abols & Hauber, 2018). In addition, across all egg models, there were 20%–30% of Oriental reed warblers that did not reject the eggs (Li, Zhang, et al., 2016), which may be inexperienced second‐year young females that had not developed a cognitive template of their own eggs yet (Lotem et al., 1995; Moskát, Bán, & Hauber, 2014).

In summary, we found that the visual signals that included both coloration and spotting were more important cues for foreign egg rejection than egg size, but egg size was a more important factor for the egg rejection response. Large eggs led to more nest desertion comparing with smaller egg size for hosts, even for Oriental reed warblers, which have a medium body size. Video samples confirmed that the accepted large model eggs may include some cases that had been recognized as foreign eggs; thus, the true egg recognition rate might be underestimated by the commonly used nest‐checking methods. In addition, we suggest that more caution should be given to assessing egg color mimicry using human vision in future egg recognition experiments.

## CONFLICT OF INTEREST

The authors declare that they have no competing interests.

## AUTHOR CONTRIBUTIONS

**Donglai Li:** Conceptualization (equal); data curation (equal); formal analysis (equal); funding acquisition (equal); investigation (equal); methodology (equal); project administration (equal); resources (equal); software (equal); supervision (equal); validation (equal); visualization (equal); writing – original draft (equal); writing – review & editing (equal). **Xiaoshuang Li:** Data curation (equal); formal analysis (equal); methodology (equal); software (equal). **Yan Zhang:** Data curation (equal); formal analysis (equal); investigation (equal). **Shuang Guan:** Data curation; formal analysis; investigation. **Yanan Ruan:** Conceptualization; investigation; project administration; resources; supervision; validation; writing – review & editing.

## Data Availability

The data used in the present study are available from the Dyrad Digital Repository (https://doi.org/10.5061/dryad.3tx95x6d6).
